# Meta-analysis of randomized controlled trials on the efficacy and safety of intracoronary administration of tirofiban for no-reflow phenomenon

**DOI:** 10.1186/1471-2261-13-68

**Published:** 2013-09-10

**Authors:** Tao Qin, Lu Xie, Meng-Hua Chen

**Affiliations:** 1 Institute of Cardiovascular Diseases, the First Affiliated Hospital of Guangxi, Medical University, Nanning, Guangxi, 530027, P. R. China; 2 Department of Physiology, School of Pre-Clinical Sciences, Guangxi Medical University, Nanning, Guangxi, 530027, P. R. China

## Abstract

**Background:**

Currently, there is still a lack of an optimal treatment for no-reflow phenomenon (NR). The aim of this simple meta-analysis was to evaluate the efficacy and safety of intracoronary (IC) administration of tirofiban compared with other conventional drugs during percutaneous coronary intervention (PCI) for NR.

**Methods:**

Systematic literature search was done from PubMed, EMBASE, Google Scholar, EBSCO, Springer and CNKI databases without language or time limitation. Randomized controlled trials were enrolled for analyzing if they investigated the treatment of IC administration of tirofiban versus other conventional drugs for NR.

**Results:**

Ten studies with 702 patients were included. Significantly, the treatment of tirofiban was more effective in improving the thrombolysis in myocardial infarction (TIMI) flow (OR 0.24, 95% CI 0.15-0.37, P < 0.00001) and reducing major adverse cardiovascular events (MACE) (OR 0.09, 95% CI 0.05-0.18, P < 0.00001). There was a trend to increase the risk of bleeding, but the data of the result did not reach the statistical significance (OR 1.44, 95% CI 0.69-3.00, P = 0.32).

**Conclusions:**

Tirofiban is more effective than conventional drugs for NR during PCI, but the potential risk of bleeding complication induced by tirofiban shouldn’t be ignored during clinical practices.

## Background

Currently, percutaneous coronary intervention (PCI) has become the most common strategy for acute coronary syndrome. No-reflow phenomenon (NR) is one of the serious complications of PCI, which could lead to poor prognosis [1-3].

The conventional pharmacological treatment for NR is intracoronary (IC) administration of vasodilators (for example, adenosine, verapamil, nitroglycerin, sodium nitroprusside, etc.) [4]. So far, there are some randomized controlled trials investigated the treatment of IC administration of tirofiban for NR. But compared with conventional drugs, the treatment of tirofiban has not been evaluated. Therefore, the aim of this article was to compare the efficacy and safety of IC administration of tirofiban with conventional drugs for NR during PCI by performing a simple meta-analysis.

## Methods

### Search strategy

Completed randomized controlled trials that investigated the efficacy and safety of IC administration of tirofiban versus conventional drug for NR during PCI were sought out by searching the electronic databases, including PubMed, EMBASE, Google Scholar, EBSCO, Springer and CNKI. Separate search strategy was developed for each database using the following keywords: “no-reflow”, “tirofiban”, “glycoprotein αb/βa inhibitors”, “intracoronary”, “randomized controlled trial” and “percutaneous coronary intervention”. The search was performed without language or time limitation. The types of articles such as comments, letters and the works that were not original reports were excluded.

### Study selection

Study was considered eligible if it met the following criteria: (i) the patients with NR during PCI were enrolled, (ii) randomly designed patients to a strategy of IC administration, either tirofiban or one of the conventional drugs, (iii) reported at least on one of following outcomes: transformation of thrombolysis in myocardial infarction (TIMI) flow after treatment, major adverse cardiovascular events (MACE) and bleeding complication. Methodological quality of the enrolled studies was assessed in relation to randomization and concealment of allocation. Quality scale was used to assess the trials: (A) true randomization and allocation concealed, and (B) process of randomization not given and concealment of allocation unclear. This approach was recommended by the Cochrane Collaboration [5].

### Data abstraction

The following information were extracted from the enrolled studies: (i) first author’s last name, publication year, (ii) study design, including the type and dosage of the IC drugs, duration of treatment, number of patients and the follow up, (iii) data of endpoints.

Outcome events were based on the definitions used in the individual trial publications. All data were independently extracted by two investigators (TQ and LX). Results were compared, and disagreements were resolved by discussion with a third investigator (MHC).

### Statistical analysis

Data were entered and analyzed using the Cochrane Collaboration Review Manager software (version 5.2). The data of outcomes were analyzed separately by indications (transformation of TIMI flow after treatment, MACE, bleeding complication). Odds ratios (OR) and 95% confidence intervals (CI) were calculated. Random-effects models were used since heterogeneity was expected among the trials. And for unifying the outcomes in forest plots, we analyzed the incidence of TIMI 0–2 flow transformation after IC treatment, which could also reflex the incidence of TIMI 3 flow, which was a signal of restoration of myocardial perfusion. An OR < 1 suggested a beneficial effect whilst an OR > 1 suggested a detrimental effect. Statistical significance was defined as a 2- sided p value < 0.05.

## Results

### Search results

**Figure 1:**
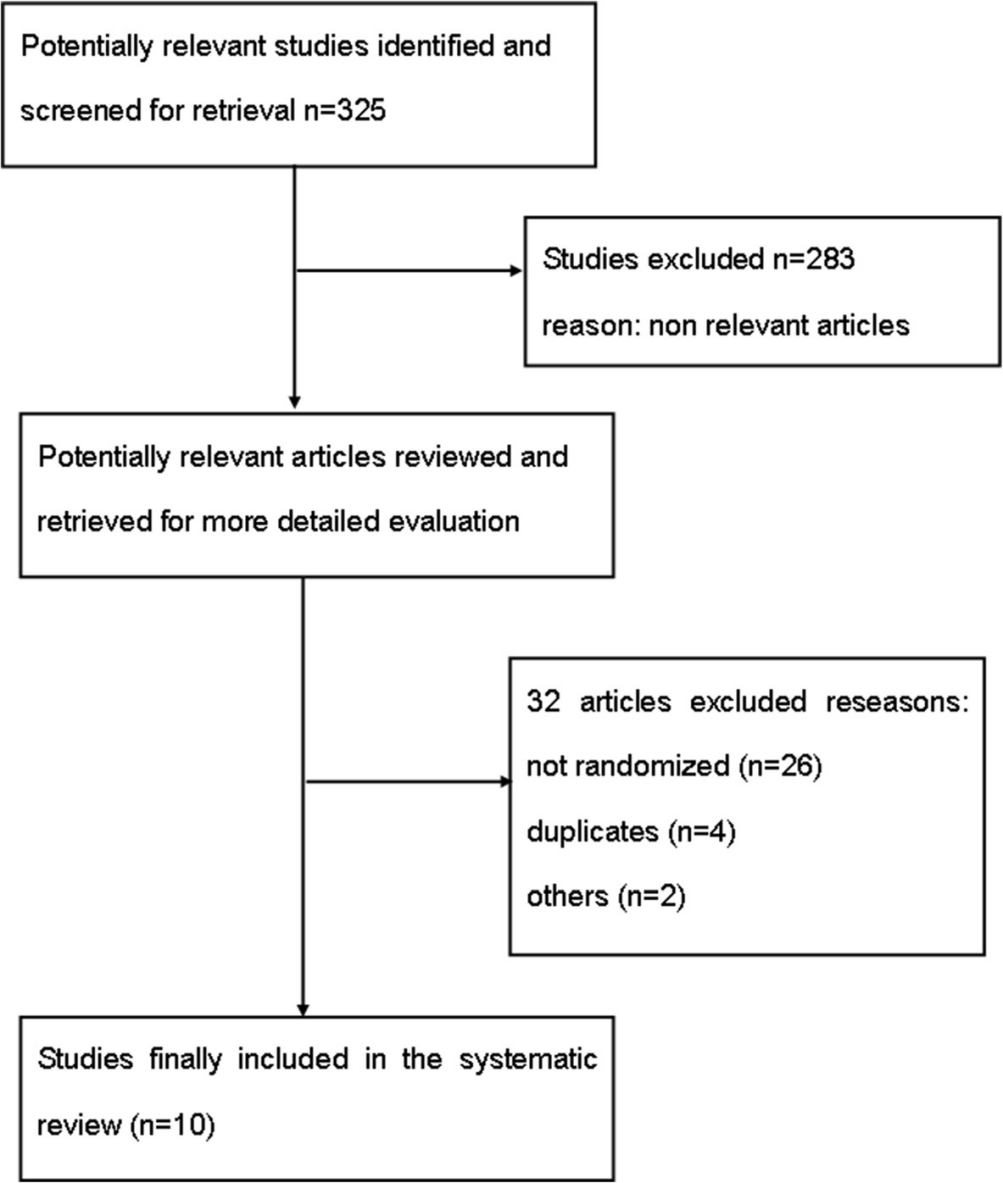
Search flow diagram of this meta-analysis.

With separate search strategy in each database, the search yielded 325 articles that were potentially pertinent. Reviewing titles and abstracts to exclude irrelevant studies, case reports, editorial comments, and reviews, 42 studies were retrieved for further consideration. Of the 42 studies, 32 studies were finally excluded mainly because they only administrated tirofiban additionally compared with the parallel control group. Ten [6-15] completed randomized trials fulfilled all the inclusion criteria and included 702 patients (Figure 1).

### Study characteristics

The enrolled studies’ characteristics are presented in Table 1. The studies published from 2007 to 2012. Described each study’s characteristic and analyzed the quality scale. All the trials mentioned randomization and the comparability of baseline were offered, but not mentioned the concealment of allocation. After the quality analyzing, all trials were evaluated B. In the control group, there were 4 trials administrated with nitroglycerin [6,9,10,12], 5 with verapamil [7,9,11,14,15] and 1 with sodium nitroprusside [13].

**Table 1:** Study design of the included randomized controlled trials

**Study**	**N(T/C)**	**Control group**		**Conventional drugs**	**Tirofiban**	**Endpoints§**	**Follow up**	Quality*
		Asp	Cpg					
**Chen2012**	27/24	√	√	Nitroglycerin 200 μg within 3 mins then IV 0.15 μg/kg•min for 36-48 h	10 μg/kg within 3 mins then IV 0.15 μg/kg•min for 36-48 h	(1)(2)(3)	30d	B
**Fu 2012**	43/43	√	√	Verapamil 1 mg within 5 mins	10 μg/kg within 5 mins then IV 0.075 μg/kg•min for 24 h	(1)	-	B
**Guan 2012**	40/40	-	-	Verapamil 1 mg within 5 mins	10 μg/kg within 5 mins then IV 0.075 μg/kg•min for 24 h	(1)	-	B
**He 2012**	28/28	√	√	Nitroglycerin 200 μg within 3 mins	10 μg/kg within 3 mins then IV 0.15 μg/kg•min for 24-48 h	(1)(2)(3)	14d	B
**Luan 2007**	45/44	-	-	Nitroglycerin 200 μg within 3 mins	10 μg/kg within 3 mins	(1)(2)(3)	7d	B
**Wang2012**	48/34	√	√	Verapamil 200 μg within 15 mins	10 μg/kg within 5 mins then IV 0.15 μg/kg•min for 38 h	(1)	-	B
**Wei 2009**	26/20	√	√	Nitroglycerin 200 μg within 3 mins then IV 0.15 μg/kg•min for 36-48 h	10 μg/kg within 3 mins then IV 0.15 μg/kg•min for 36-48 h	(1)(2)(3)	30d	B
**Wu 2012**	36/36	√	√	Sodium nitroprusside 0.9 μg/kg within 3 mins	10 μg/kg within 3 mins then IV 0.15 μg/kg•min for 24 h	(1)(2)(3)	14d	B
**Zhang 2010**	46/49	√	√	Verapamil 1 mg within 5 mins	10 μg/kg within 5 mins then IV 0.075 μg/kg•min for 24 h	(1)	-	B
**Zhang 2012**	21/24	√	√	Verapamil 200 μg within 3 mins	10 μg/kg within 3 mins then IV 0.15 μg/kg•min for 36-48 h	(1)(2)(3)	30d	B

§ (1) transformation of TIMI flow, (2) MACE, (3) bleeding complication. *Quality scale: A, true randomization and allocation concealed; B, process of randomization not given and concealment of allocation unclear. Asp, aspirin; Cop, clopidogrel.

### Quantitative data synthesis

**Figure 2:**
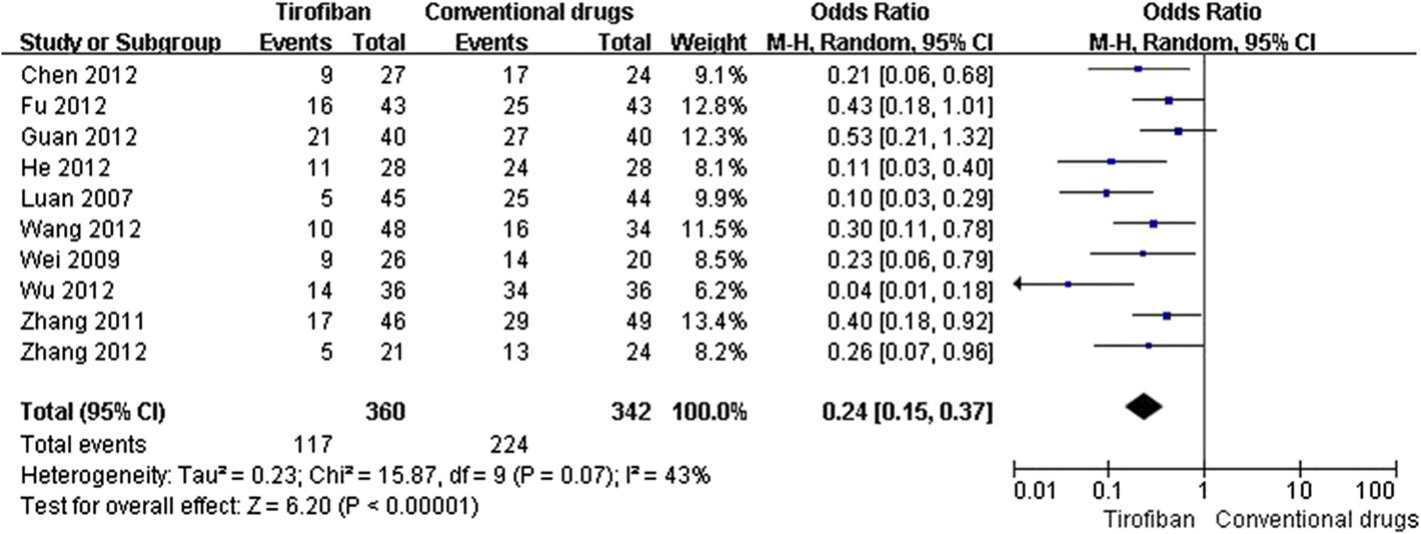
Forest plot of OR for TIMI flow transformation.

**Figure 3:**
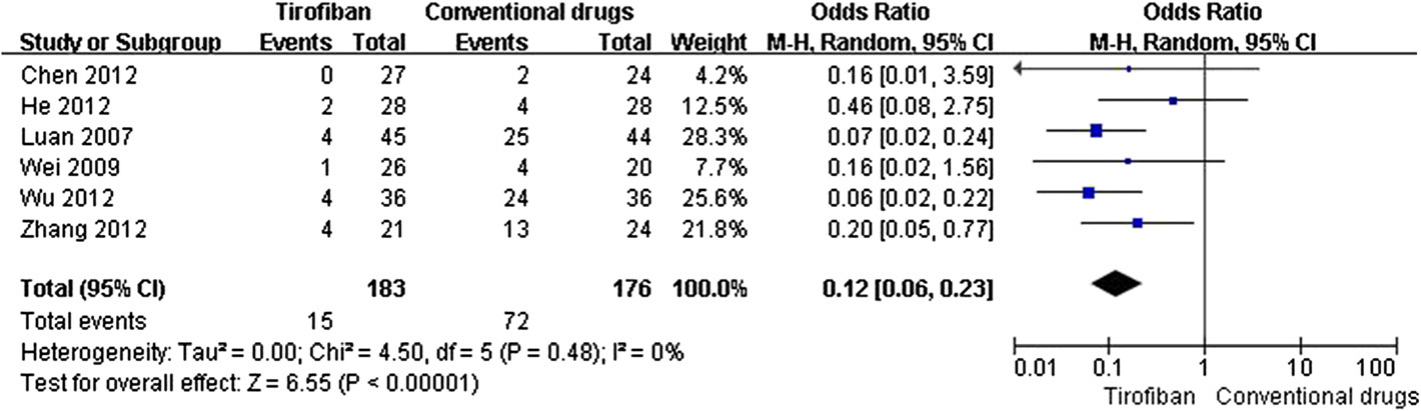
Forest plot of OR for MACE.

**Figure 4:**
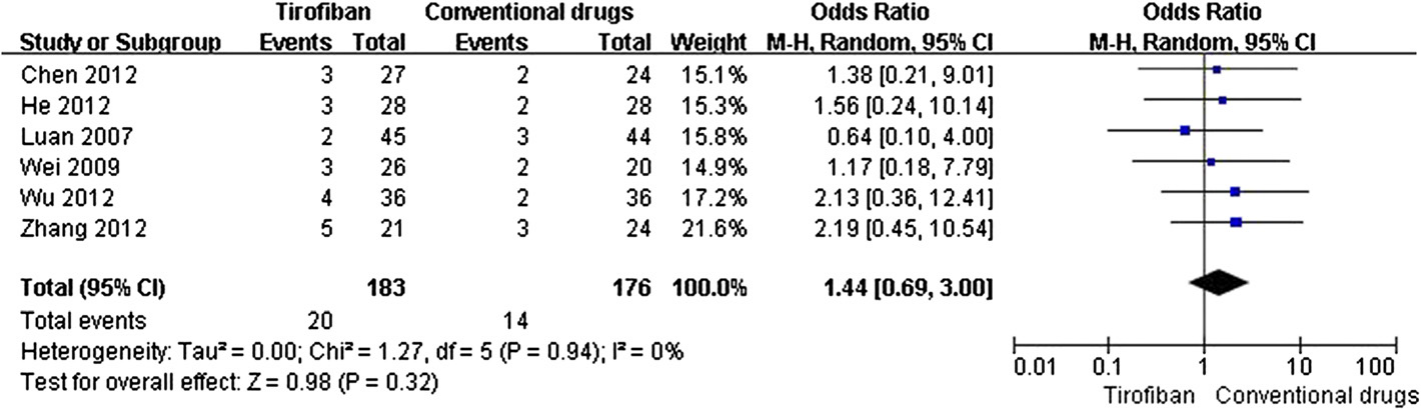
Forest plot of OR for the risk of bleeding complication.

According to the data analysis, all the enrolled trials (n = 702) investigated the transformation of TIMI flow after the IC pharmacological treatment and the results found that tirofiban was more effective in improving the TIMI flow (OR 0.24, 95% CI 0.15-0.37, P < 0.00001, Figure 2). Furthermore, tirofiban significantly reduced the MACE (OR 0.09, 95% CI 0.05-0.18, P < 0.00001, Figure 3), but had a tendency to increase the risk of bleeding (OR 1.44, 95% CI 0.69-3.00, P = 0.32, Figure 4) in 6 of the 10 trials (n = 359) [6,9,10,12,13,15].

**Figure 5:**
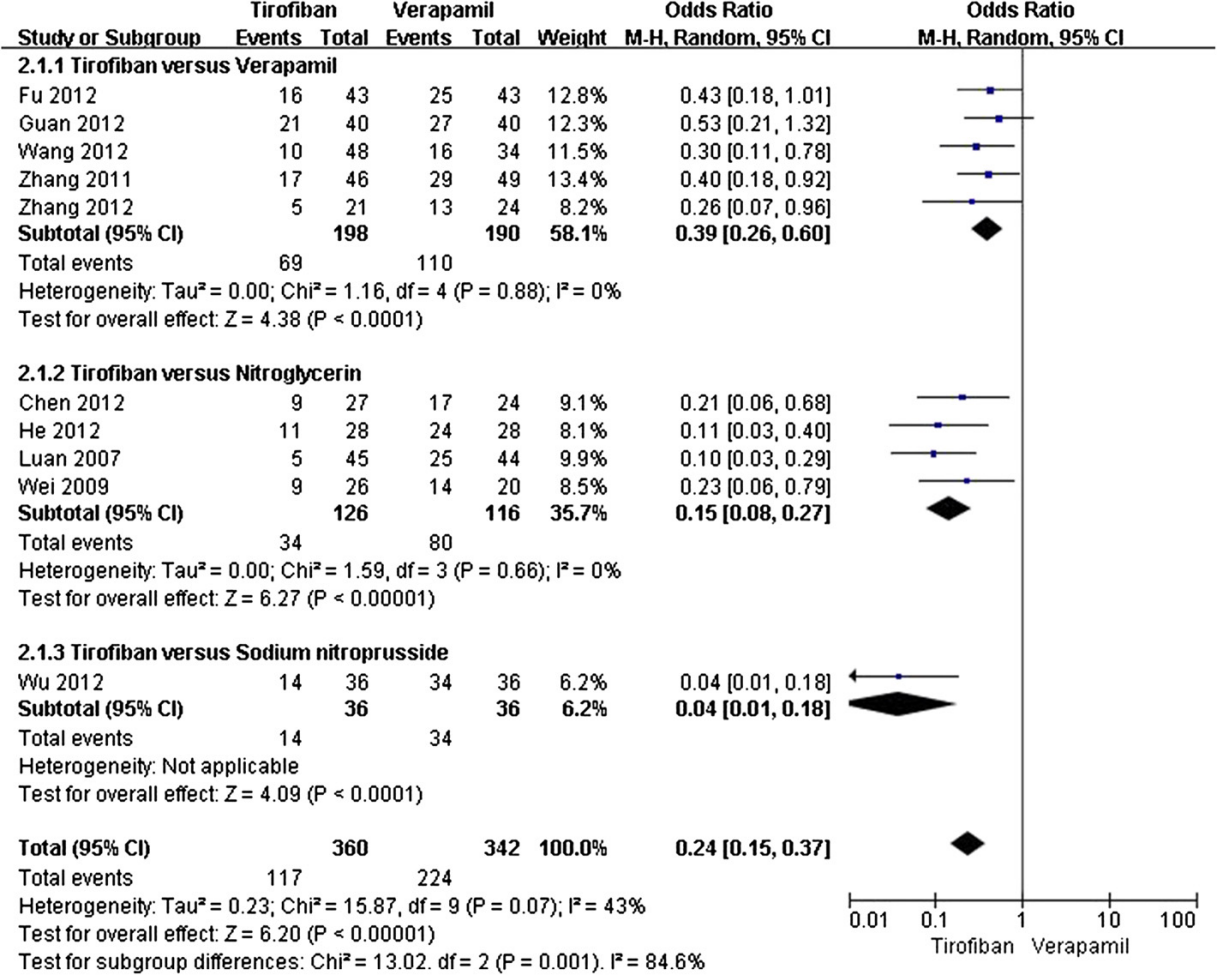
Forest plot of OR for TIMI, compared tirofiban with each kind of conventional drugs.

Specifically, we compared tirofiban with each kind of conventional drugs for a further analysis of the transformation of TIMI flow. And it also indicated that tirofiban had its benefit of improving the coronary flow for NR (OR 0.24, 95% CI 0.15-0.37, P = 0.001, Figure 5).

## Discussion

NR could be defined as the persistence of reduced flow and regional myocardial dysfunction after the removal of an experimental epicardial coronary occlusion [16].

So far, the precise mechanisms of NR have not been fully clarified. The optimal therapy for NR is still being explored. Some studies [17-19] suggested that the dysfunction of coronary microcirculation perfusion was the central mechanism of NR. And it would not occur until the lesion of coronary microvascular endothelium to a certain extent. It was a dynamic and persistent procedure. Once the phenomenon occurred, the inflammation and lesion of coronary microvascular endothelium would be aggravated and the effect would sustain for weeks. A case report [20] showed that the phenomenon could be improved by taking appropriate intervention measures before the lesion. Restoration of myocardial perfusion rapidly could be achieved by removing the microvascular obstruction and recovering the antegrade coronary flow of occlusive vessel, and it has become a key of the treatment for NR [21].

For NR, the mechanism of conventional drugs was mainly for expanding the coronary vessel, which might be beneficial to allowing the formed microthrombus to get through the microvascular network and removing the coronary occlusion. On the basis of the mechanisms, conventional drugs could not inhibit the sustained thrombi caused by platelet aggregation when balloon was dilating, which limited the effect [22,23]. The effects of these vasodilators in patients with NR were contradictory and could not be sustained by large scale clinical evidence [24-29].

Platelet aggregation plays an important role in the formation of embolization. Glycoprotein ΙΙb/ΙΙΙa inhibitors (GPI) block the final pathway of platelet aggregation, combine with the glycoprotein ΙΙb/ΙΙΙa receptors selectively and inhibit the thrombinogen I competitively. And also, GPI could inhibit the activation, adhesion and aggregation of platelet. The pharmacological mechanisms of GPI were contributed to the formation of platelet thrombi, restoration the antegrade coronary flow of occlusive vessel and reducing the incidence of the ischemia event [30,31]. From the recent researches, GPI has its obvious advantages in inhibiting the formation of platelet thrombus, but bleeding event was the main complication [31,32]. Tirofiban is one kind of GPI, which with high selectivity and short-acting pharmacological mechanism [33]. During PCI, IC administration of tirofiban might increase the local drug concentration and improve the coronary flow. Considering the particular mechanism, tirofiban selectively blocks the final pathway of the platelet aggregation, which might contribute to the improving TIMI flow. And the short half-life might relative to the reducing of MACE.

Also, the bleeding event needs to be considered. By analyzing the enrolled trials of this article, 9 of the 10 trials had the administration of tirofiban by intravenous (IV) infusion after PCI with some degrees. Chen and Wei administrated both group with IV infusion [6,12], while the other seven trials only with the tirofiban group [7-9,11-15]. Although it didn’t reach the statistical significance, an increasing trend of bleeding was observed comparing with the conventional drugs. One of the possible explanations might be that IV infusion of tirofiban increased the drug concentration in the system and decreased the function of platelet which led to the bleeding complication. Kimmelstiel et al. [34] referred that the half-life of tirofiban was 2 hours, and the function of platelet could recover 89% of the baseline and the prothrombin time recovered completely after 2–4 hours of the drug withdrawal. In a study by Zhang WZ et al. [35] of IC administration tirofiban for NR, if the effect was not satisfied during the PCI, then continually IV infused for 24-hour post-operation could improve the antegrade coronary flow of occlusive vessel. But from a meta-analysis by Geeganage C et al. [36], it suggested that the additional IV administration of GPI increased the bleeding event. However, the administration of post-operative IV infusion of tirofiban for NR, whether or how it should be, needs to be confirmed by further clinical investigations.

### Study limitations

Though TIMI is a classical indicator of reperfusion during PCI [37-39], it doesn’t mean that TIMI 3 flow represents a normal myocardial perfusion [40]. In other words, myocardial blush grade (MBG) 0–1 might occur in the patients with TIMI 3 flow during PCI. It had been found that MBG was an independent predictor of long-term mortality and could be used to describe the effectiveness of myocardial reperfusion [41]. Van’t Hof et al. [42] proposed that the angiographic definition of successful reperfusion should include both TIMI 3 flow as well as MBG 2 or 3. Moreover, Stone et al. [43] suggested that MBG could be used to stratify prognosis of survival in high risk patients achieving TIMI 3 flow after intervention. Theoretically, MBG is superior to TIMI when assessing the myocardial perfusion during PCI. Unfortunately, we didn’t use MBG as an indicator in the present study, because only one of the ten enrolled studies provided the data of TIMI as well as MBG in the comparison of trofiban versus verapamil [7].

As all the enrolled trials’ quality scales were B and with small sample data, the bias should not be ignored. Also, the condition of patients, the time and dosage of different administrations and the different selections of the conventional drugs might have influenced the outcomes. Therefore, it needs further powerful randomized trials to investigate the treatment.

## Conclusions

The treatment of IC administration of tirofiban is more effective than the conventional drugs for NR, but the potential risk of bleeding complication induced by the drug shouldn’t be ignored during clinical practices. From this meta-analysis, it suggested that the safety of IC administration of tirofiban for NR should be fully considered the individuation of patients and balanced the efficacy and the hazard.

## Competing interests

The authors declare that they have no competing interests.

## Authors’ contributions

TQ conceived the study, participated in the design, collected the data, and drafted the manuscript. LX participated in the design, collected the data, performed statistical analyses and drafted the manuscript. MHC conceived the study, participated in the design, and helped to draft the manuscript. All authors read and approved the final manuscript.

## Authors’ information

Tao Qin and Lu Xie are co-first author.

## Pre-publication history

The pre-publication history for this paper can be accessed here:

http://www.biomedcentral.com/1471-2261/13/68/prepub
